# Supplementary material for: Synthesis and Physicochemical Properties of 2,7-Disubstituted Phenanthro[2,1-b:7,8-b’]dithiophenes
Source: Molecules. 2020 Aug 24;25(17):3842. doi: 10.3390/molecules25173842 (PMC7504374; doi:10.3390/molecules25173842)
Supplement: Supplementary file 1 [file molecules-25-03842-s001.pdf]

Supplementary Materials

Synthesis and Physicochemical Properties of 2,7-Disubstituted Phenanthro[2,1-*b*:7,8-*b*']dithiophenes

Zhenfei Ji[1], Zeliang Cheng[1],

Hiroki Mori[2], and Yasushi Nishihara[2,*]

[1] Graduate School of Natural Science and Technology, Okayama University, 3-1-1 Tsushimanaka, Kita-ku, Okayama 700-8530, Japan; puf77a9l@s.okayama-u.ac.jp (Z.J.); pywp1wa6@s.okayama-u.ac.jp (Z.C.)

[2] Research Institute for Interdisciplinary Science, Okayama University, 3-1-1 Tsushimanaka, Kita-ku, Okayama 700-8530, Japan; h-mor@okayama-u.ac.jp (H.M.); ynishiha@okayama-u.ac.jp (Y.N.)

* Correspondence: ynishiha@okayama-u.ac.jp; Tel.: +81-86-251-7855

The molecular geometries were optimized using density functional theory (DFT) at the B3LYP/6-31G (d) level using Gaussian 09, Revision D. 01.

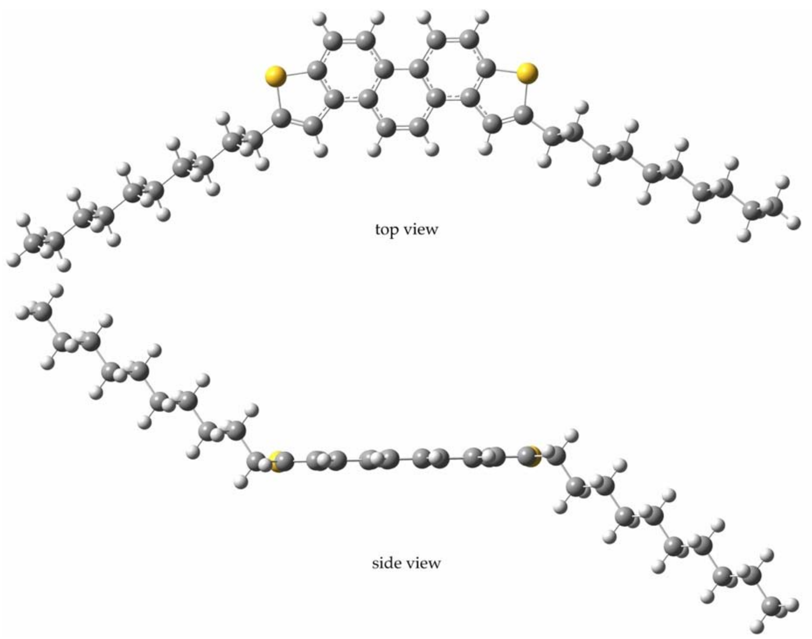

Figure S1. Optimized molecular structure of C_{10}-PDT-2.

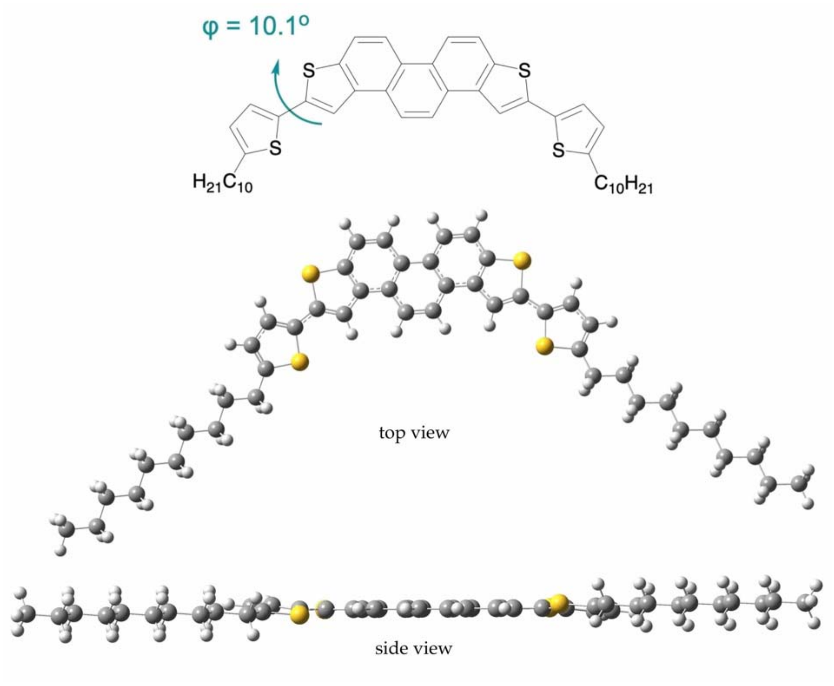

Figure S2. Optimized molecular structure and computed dihedral angle of Th1-PDT-2.

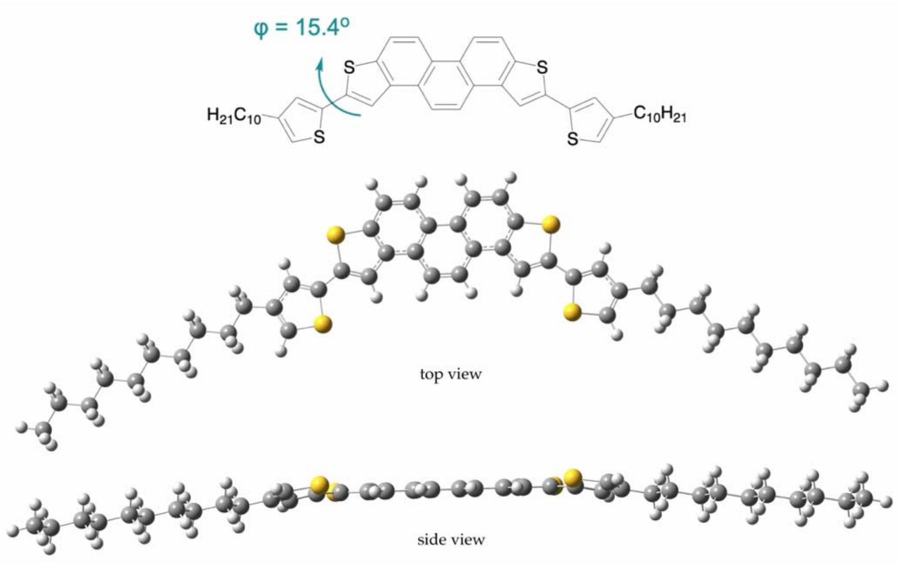

Figure S3. Optimized molecular structure and computed dihedral angle of Th2-PDT-2.

Table S1. Calculated bond lengths of Th1-PDT-2 and Th2-PDT-2 using the DFT with the B3LYP/6-31(d) level.

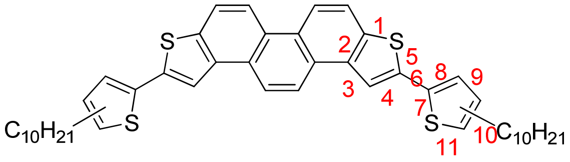

Bond lengths (Å)	1	2	3	4	5	6	7	8	9	10	11
Th1-PDT-2	1.75	1.41	1.43	1.37	1.77	1.45	1.76	1.38	1.42	1.37	1.75
Th2-PDT-2	1.75	1.41	1.43	1.37	1.76	1.45	1.76	1.37	1.43	1.37	1.74

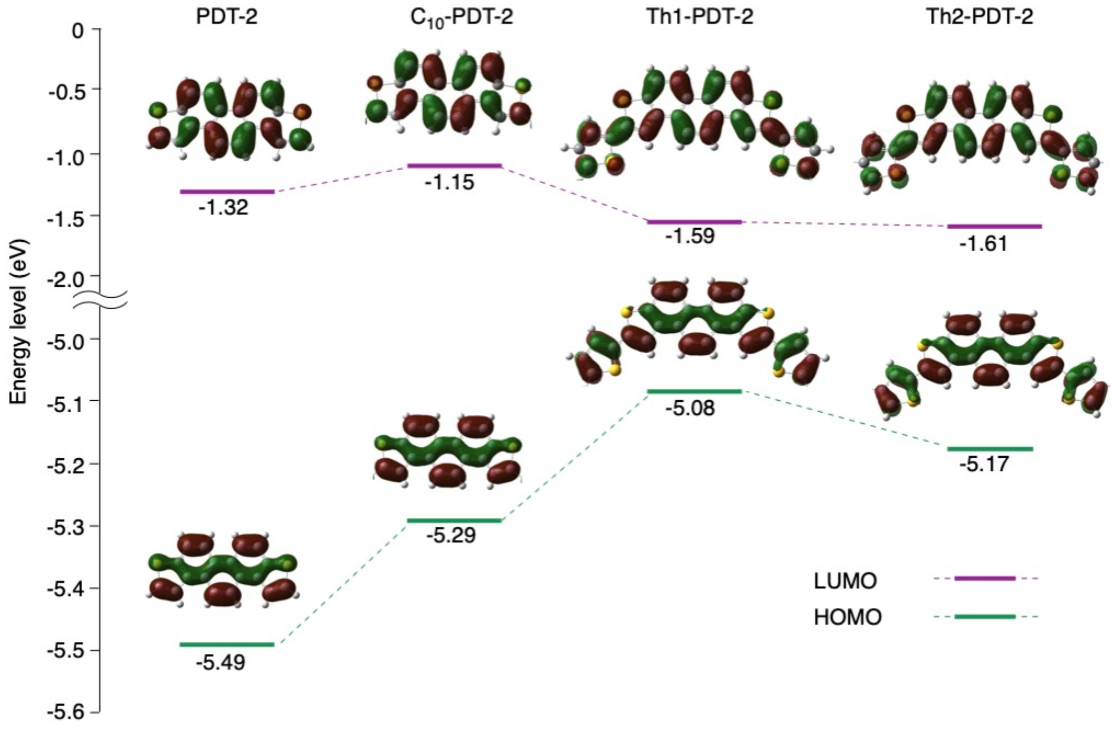

Figure S4. Calculated LUMO and HOMO distributions of PDT-2, C_{10}-PDT-2, Th1-PDT-2, and Th2-PDT-2.

Table S2. Solubility of PDT-2 derivatives in chloroform and toluene at room temperature.

Compounds	Solubility in chloroform (g/L)	Solubility in toluene (g/L)
C_8-PDT-2	5.00	0.50
C_{10}-PDT-2	3.33	0.47
C_{12}-PDT-2	1.25	0.25
C_{13}-PDT-2	1.00	0.12
C_{14}-PDT-2	0.77	0.11
Th1-PDT-2	0.15	0.07
Th2-PDT-2	2.67	2.00

Table S3. Absorption bands of C_{10}-PDT-2 in chloroform and assigned transitions according to time dependent density functional theory (TD-DFT) calculations (PCM, chloroform/B3LYP/6-31G(d)).

λ_{exp} (nm)	λ (oscillator strength)	assignment[a]
365	495 (f = 0.0000)	HOMO → LUMO
	414 (f = 0.0000)	HOMO → LUMO+1
		HOMO-1 → LUMO
		HOMO-2 → LUMO
	366 (f = 0.0000)	HOMO-1 → LUMO
		HOMO-2 → LUMO
339	342 (f = 0.6486)	HOMO → LUMO
		HOMO-1 → LUMO+1
324	333 (f = 0.0044)	HOMO-1 → LUMO
		HOMO → LUMO+1
304	297 (f = 0.0046)	HOMO-2 → LUMO
	289 (f = 0.1720)	HOMO-1 → LUMO
		HOMO → LUMO+1
276	280 (f = 0.4097)	HOMO-2 → LUMO+2
		HOMO-1 → LUMO+1
	270 (f = 0.1520)	HOMO-1 → LUMO+1
		HOMO-2 → LUMO+1
257	254 (f = 0.2567)	HOMO → LUMO+3
	252 (f = 1.2477)	HOMO-1 → LUMO+2

[a] Major contributions.

Table S4. Absorption bands of Th1-PDT-2 in chloroform and assigned transitions according to time dependent density functional theory (TD-DFT) calculations (PCM, chloroform/B3LYP/6-31G(d)).

λ_{exp} (nm)	λ (oscillator strength)	assignment[a]
384	407 (f = 1.2884)	HOMO → LUMO
365	360 (f = 0.4835)	HOMO-1 → LUMO
		HOMO → LUMO+1
	345 (f =0 .3924)	HOMO-1 → LUMO+1
293	300 (f = 0.3637)	HOMO-3 → LUMO
		HOMO-2 → LUMO+1
	286 (f = 0.2605)	HOMO-1 → LUMO+2
263	259 (f = 0.7836)	HOMO-2 → LUMO+2

[a] Major contributions.

Table S5. Absorption bands of Th2-PDT-2 in chloroform and assigned transitions according to time dependent density functional theory (TD-DFT) calculations (PCM, chloroform/B3LYP/6-31G(d)).

λ_{exp} (nm)	λ (oscillator strength)	assignment[a]
380	400 (f = 1.2615)	HOMO → LUMO
362	353 (f = 0.3728)	HOMO-1 → LUMO
		HOMO → LUMO+1
	337 (f = 0.3689)	HOMO-1 → LUMO+1
292	296 (f = 0.3494)	HOMO-3 → LUMO
		HOMO-1 → LUMO+2
	281 (f = 0.2918)	HOMO-3 → LUMO
		HOMO-1 → LUMO+2
263	259 (f = 0.7712)	HOMO-2 → LUMO+2

[a] Major contributions.

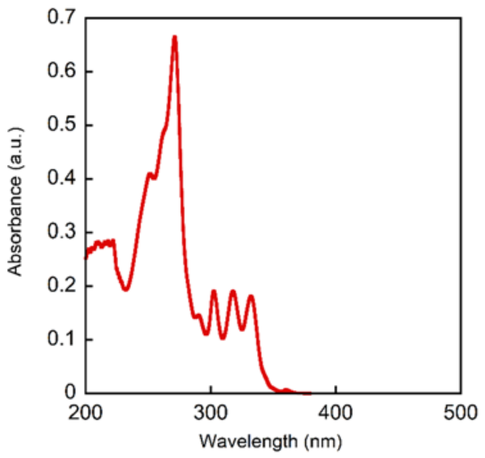

Figure S5. UV-vis absorption spectra of parent PDT-2 in solution

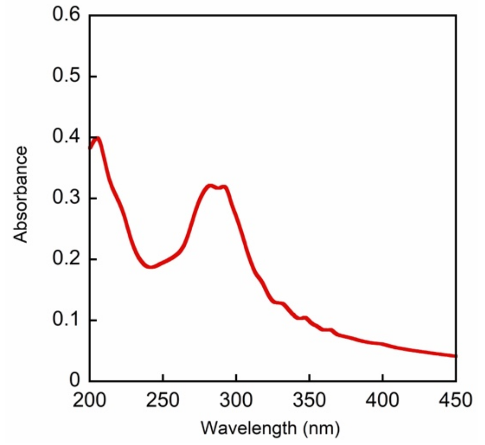

Figure S6. UV-vis absorption spectra of parent PDT-2 in thin film

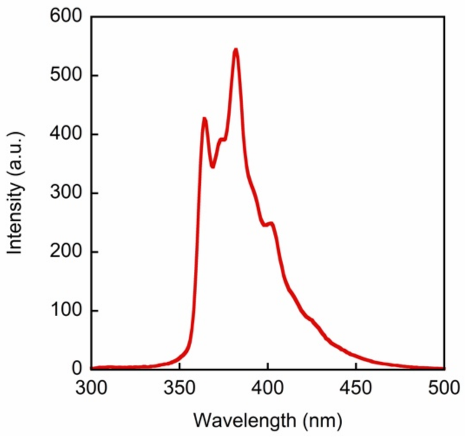

Figure S7. Fluorescence spectra of parent PDT-2 in solution

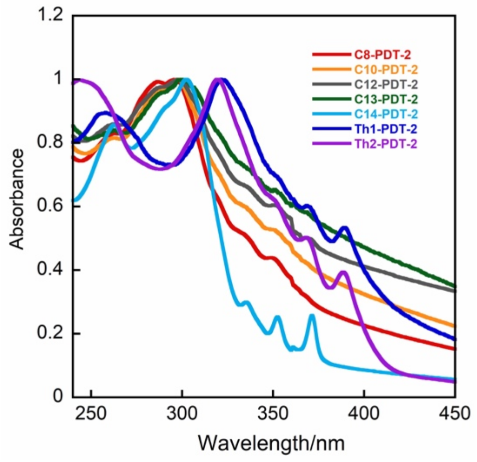

Figure S8. UV-vis absorption spectra of PDT-2 derivatives in thin film.

Charts of NMR Spectra

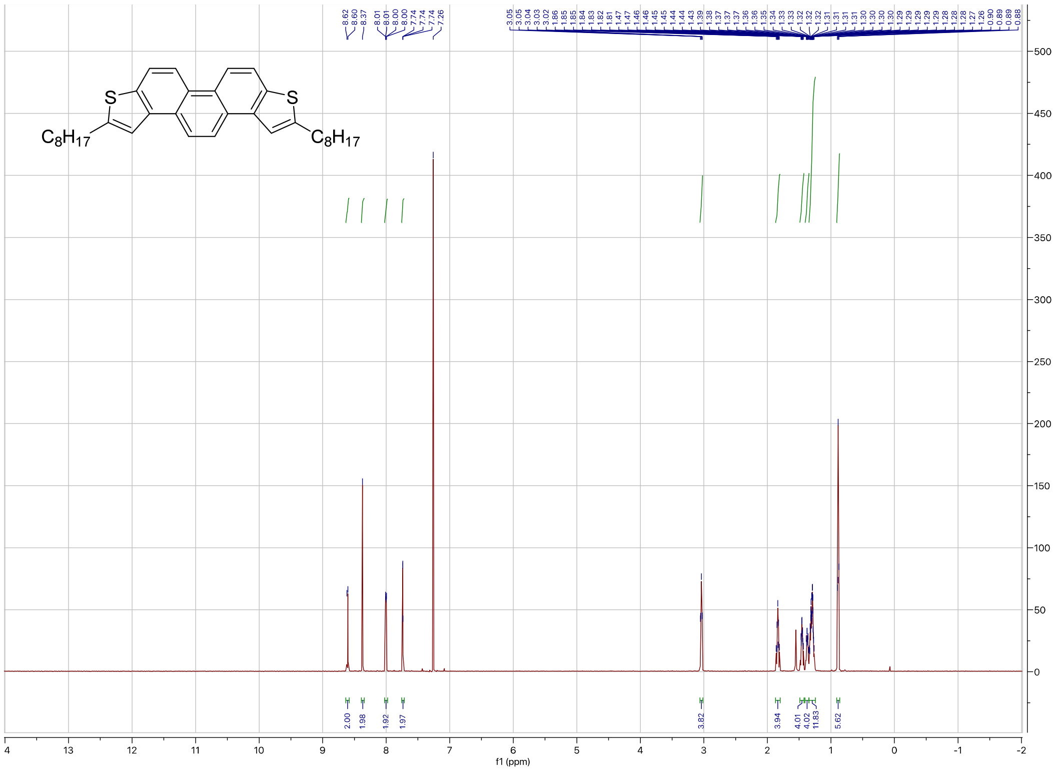

^{1}H NMR (600 MHz) spectrum of C_8-PDT-2 ($CDCl_3$, rt).

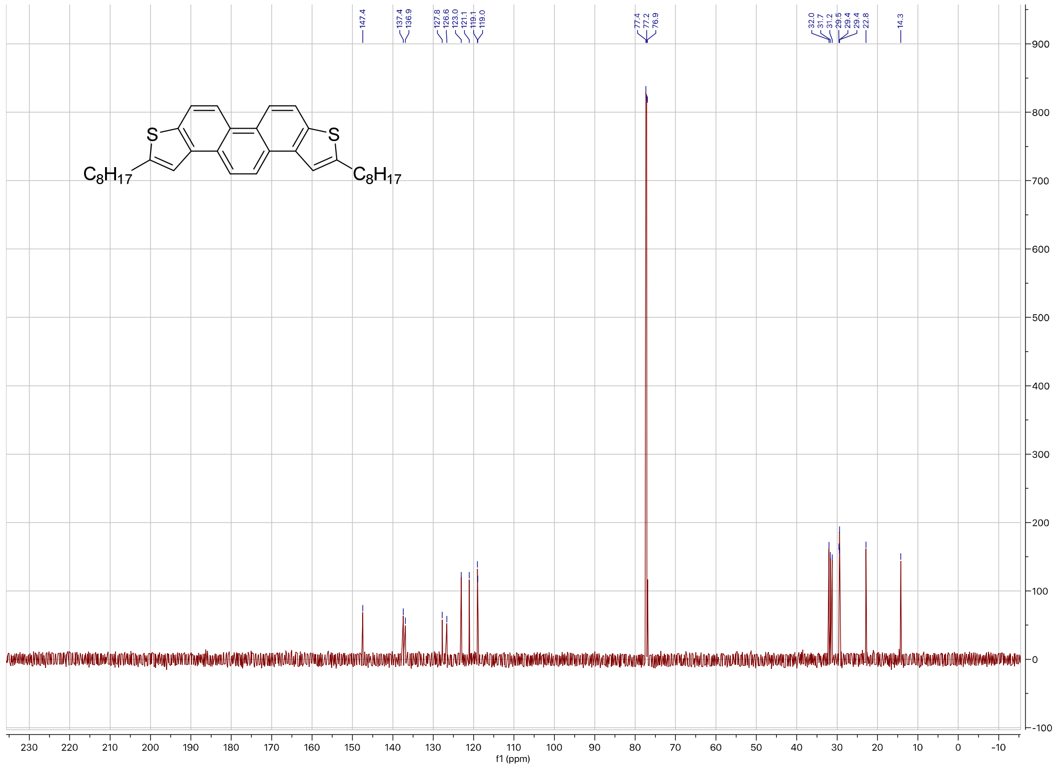

$^{13}C\{^{1}H\}$ NMR (150 MHz) spectrum of C_8-PDT-2 ($CDCl_3$, rt).

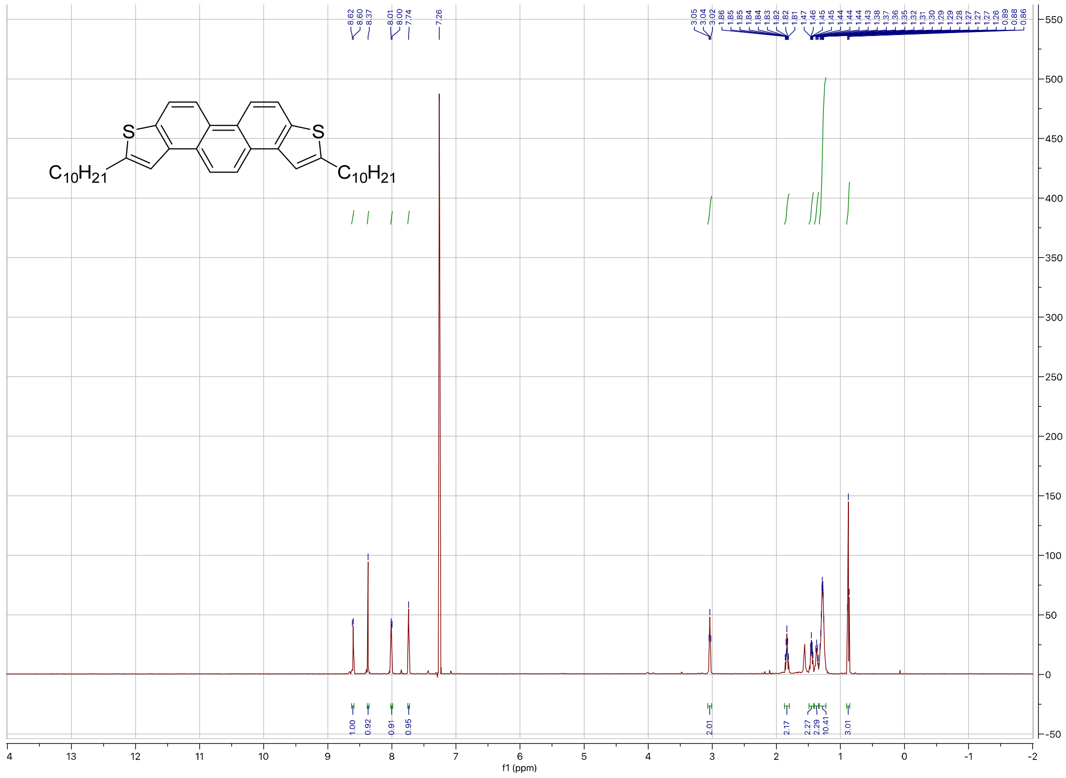

^{1}H NMR (600 MHz) spectrum of C_{10}-PDT-2 ($CDCl_3$, rt).

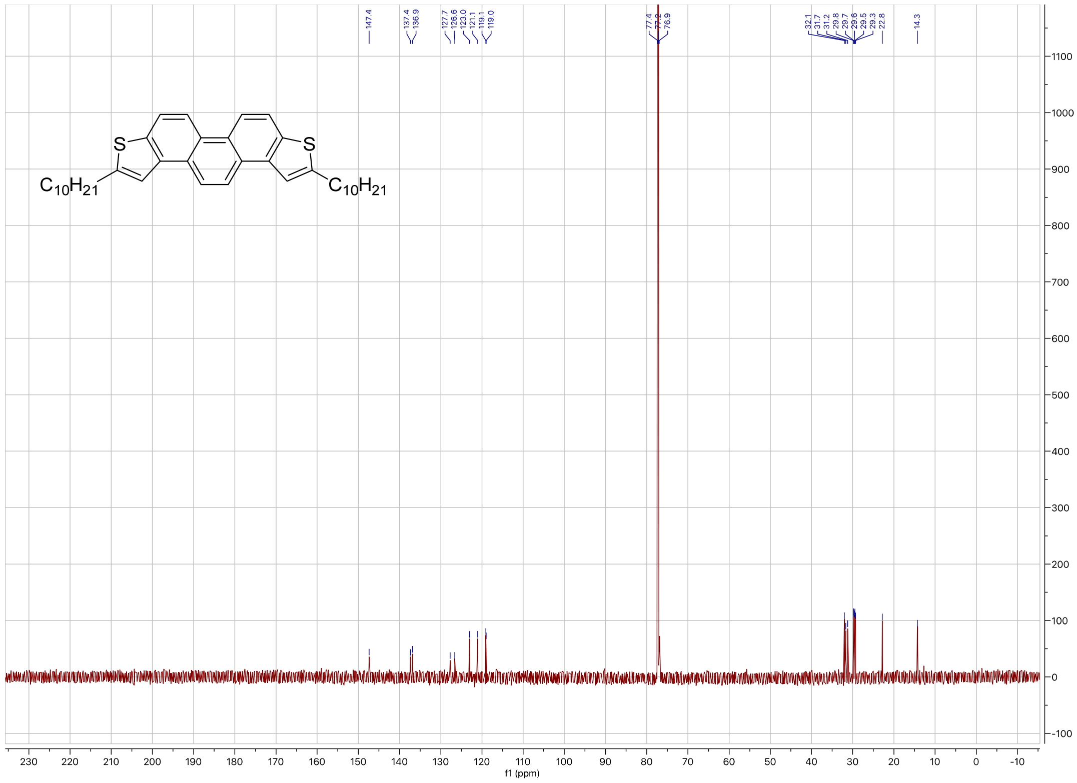

$^{13}C\{^{1}H\}$ NMR (150 MHz) spectrum of C_{10}-PDT-2 ($CDCl_3$, rt).

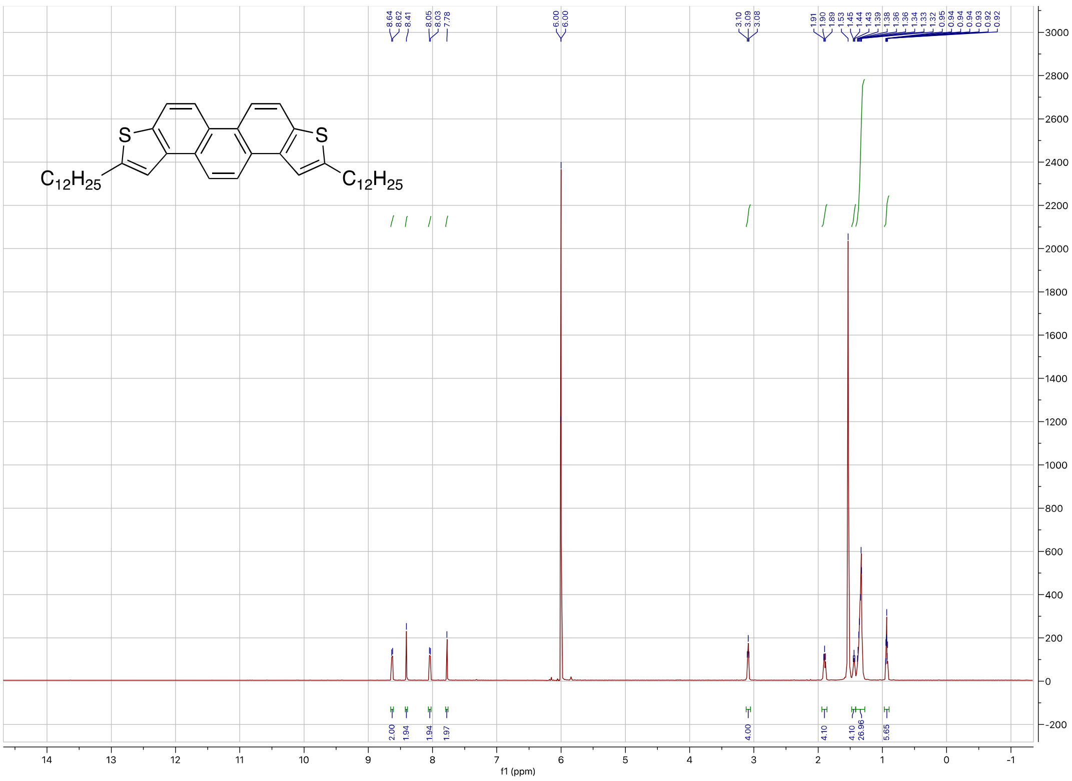

^{1}H NMR (600 MHz) spectrum of C_{12}-PDT-2 (TCE-d_2, 80 °C).

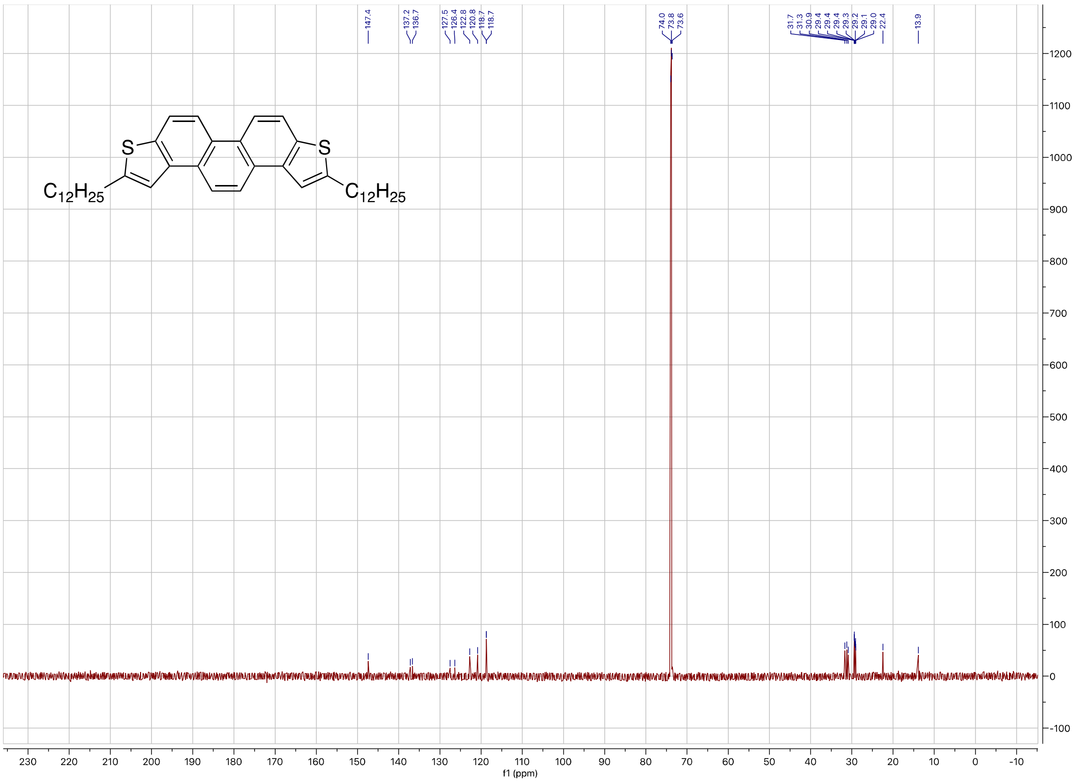

^{13}C{^{1}H} NMR (150 MHz) spectrum of C_{12}-PDT-2 (TCE-d_2, 80 °C).

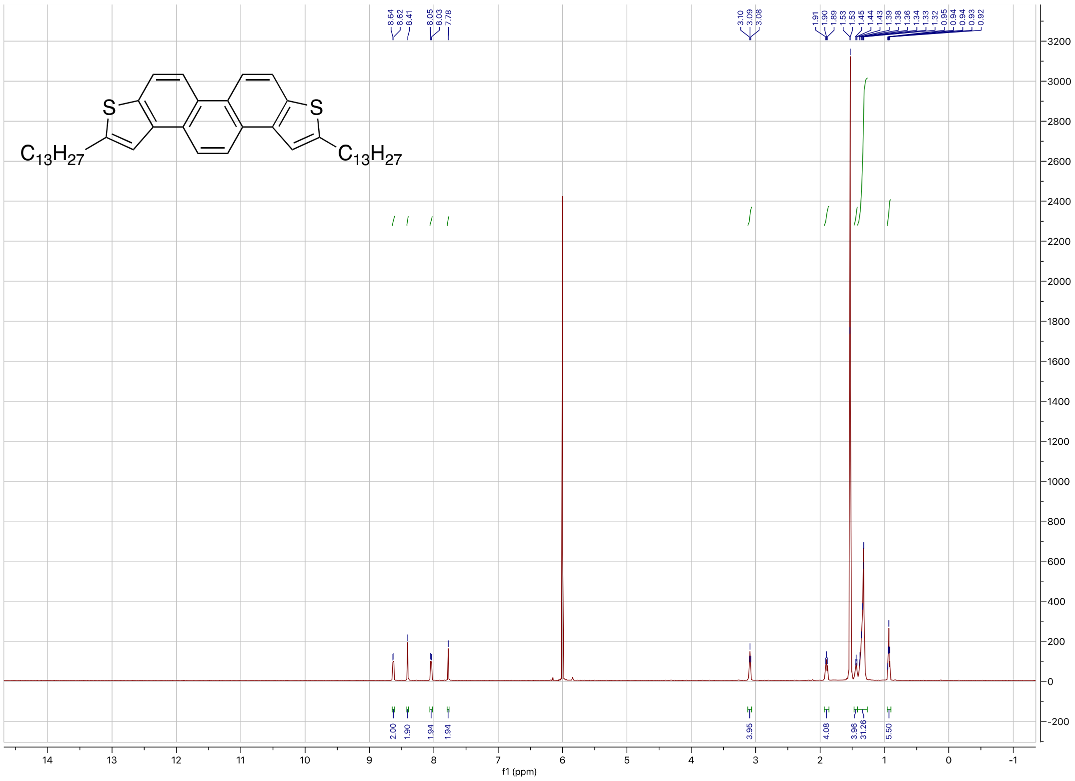

^{1}H NMR (600 MHz) spectrum of C_{13}-PDT-2 (TCE-d_2, 80 °C).

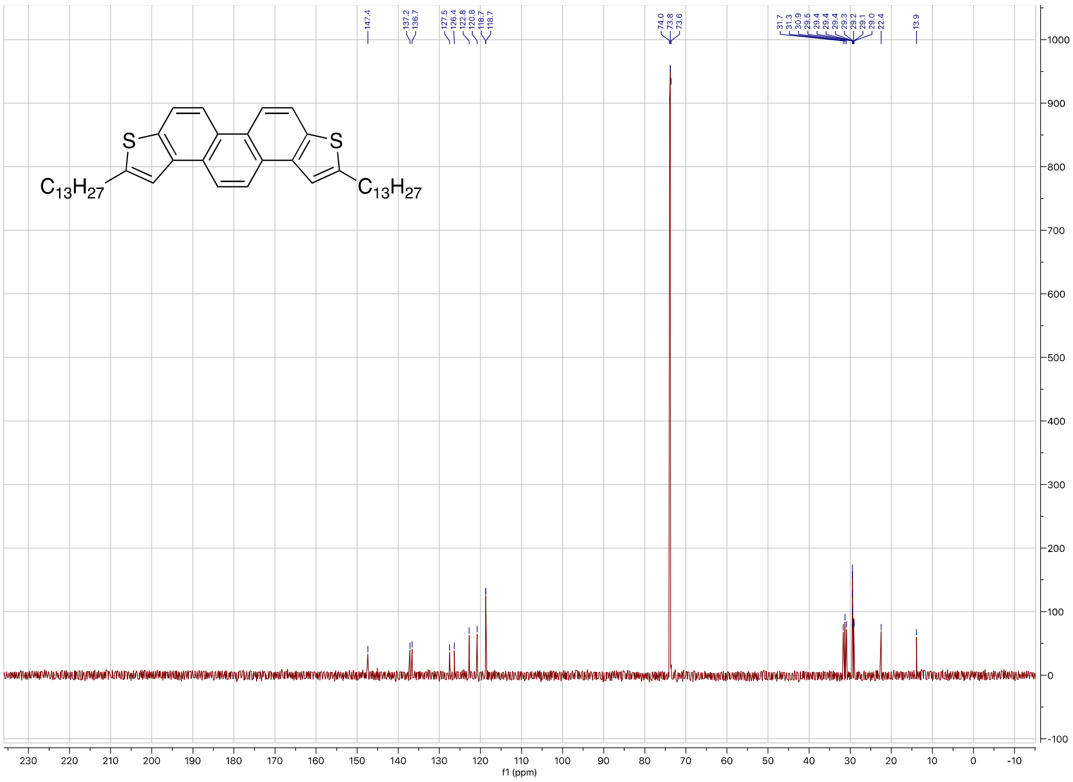

^{13}C{^{1}H} NMR (150 MHz) spectrum of C_{13}-PDT-2 (TCE-d_2, 80 °C).

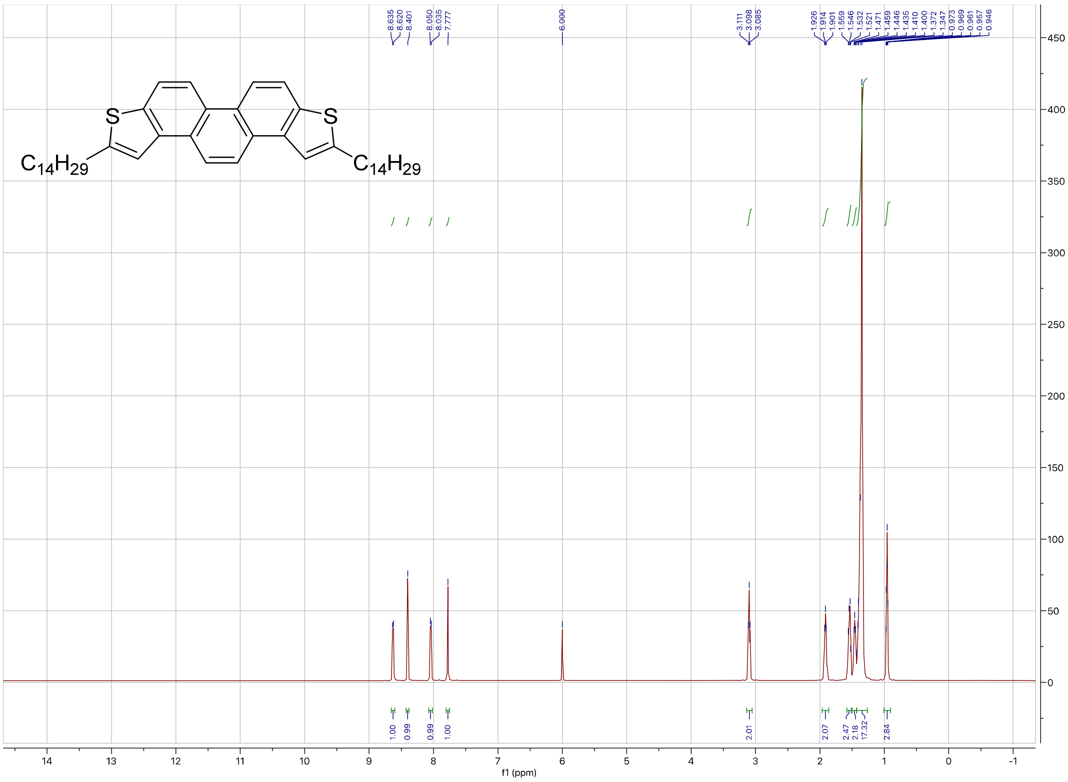

^{1}H NMR (600 MHz) spectrum of C_{14}-PDT-2 (TCE-d_2, 80 °C).

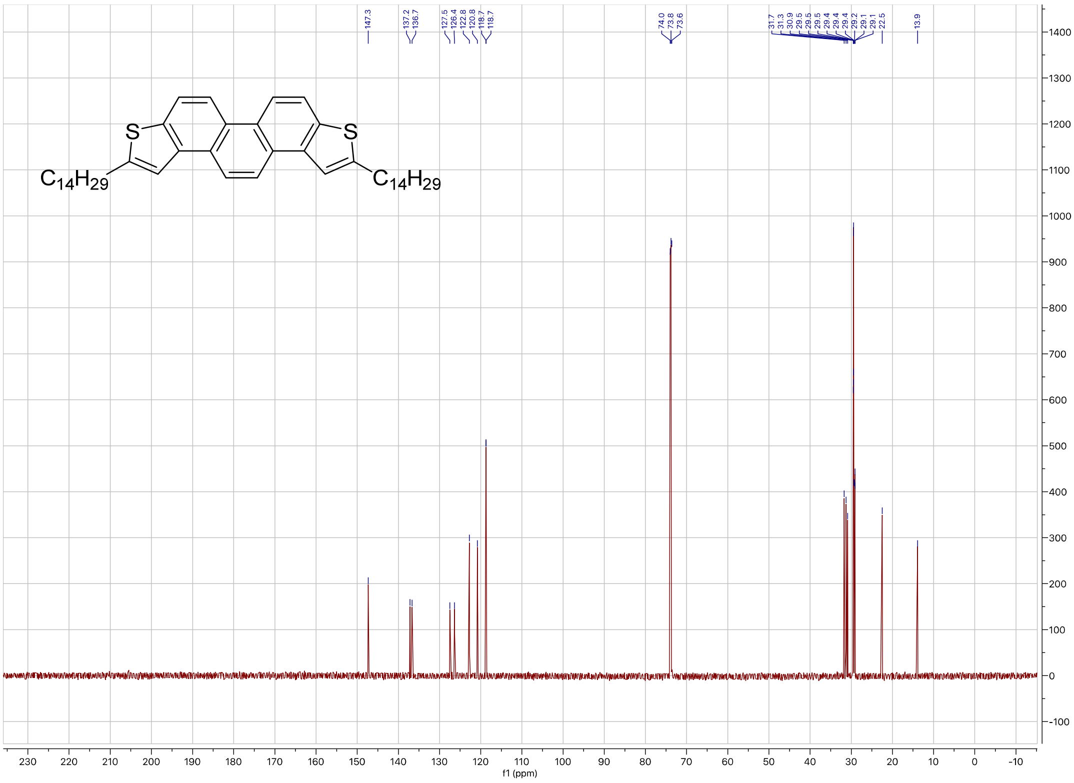

^{13}C{^{1}H} NMR (150 MHz) spectrum of C_{14}-PDT-2 (TCE-d_2, 80 °C).

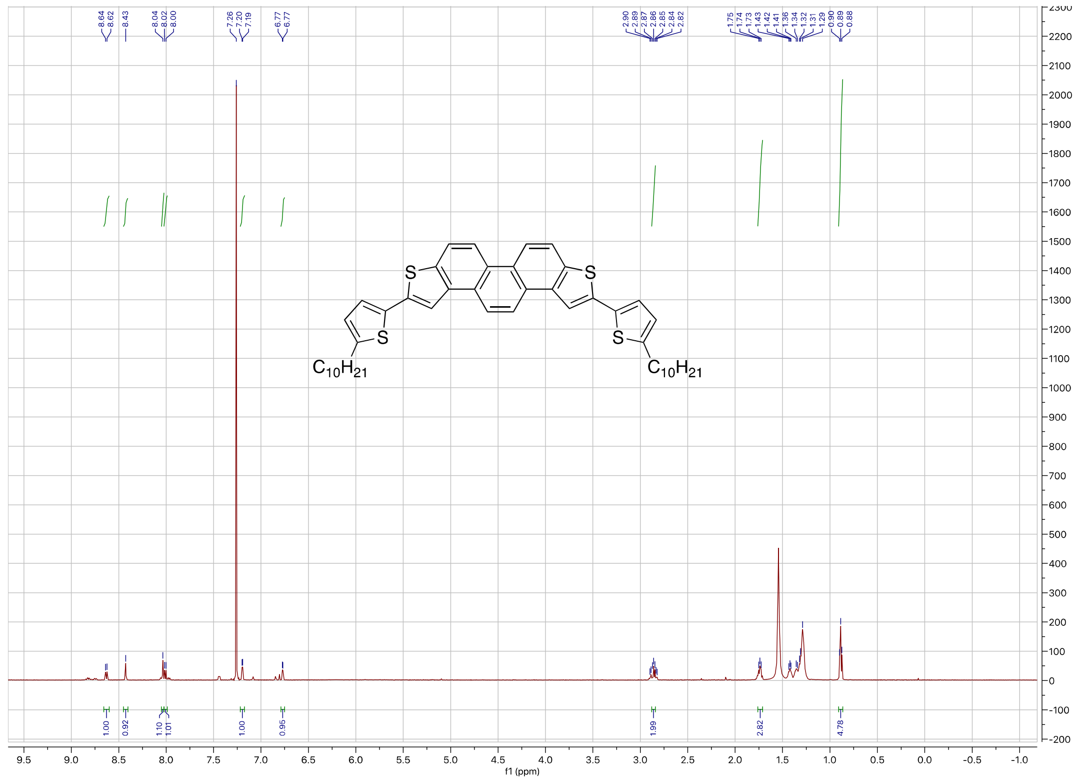

^{1}H NMR (600 MHz) spectrum of Th1-PDT-2 ($CDCl_3$, rt).

The $^{13}C\{^1H\}$ NMR spectrum of Th1-PDT-2 could not be measured due to its low solubility.

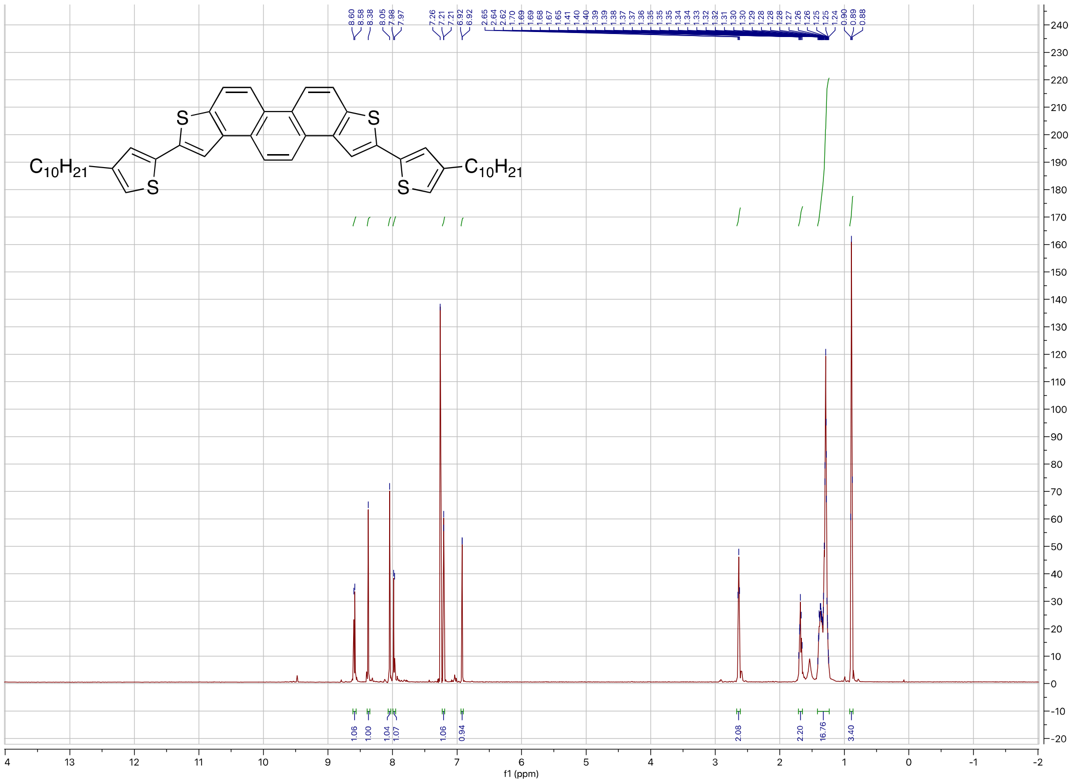

^{1}H NMR (600 MHz) spectrum of Th2-PDT-2 ($CDCl_3$, rt).

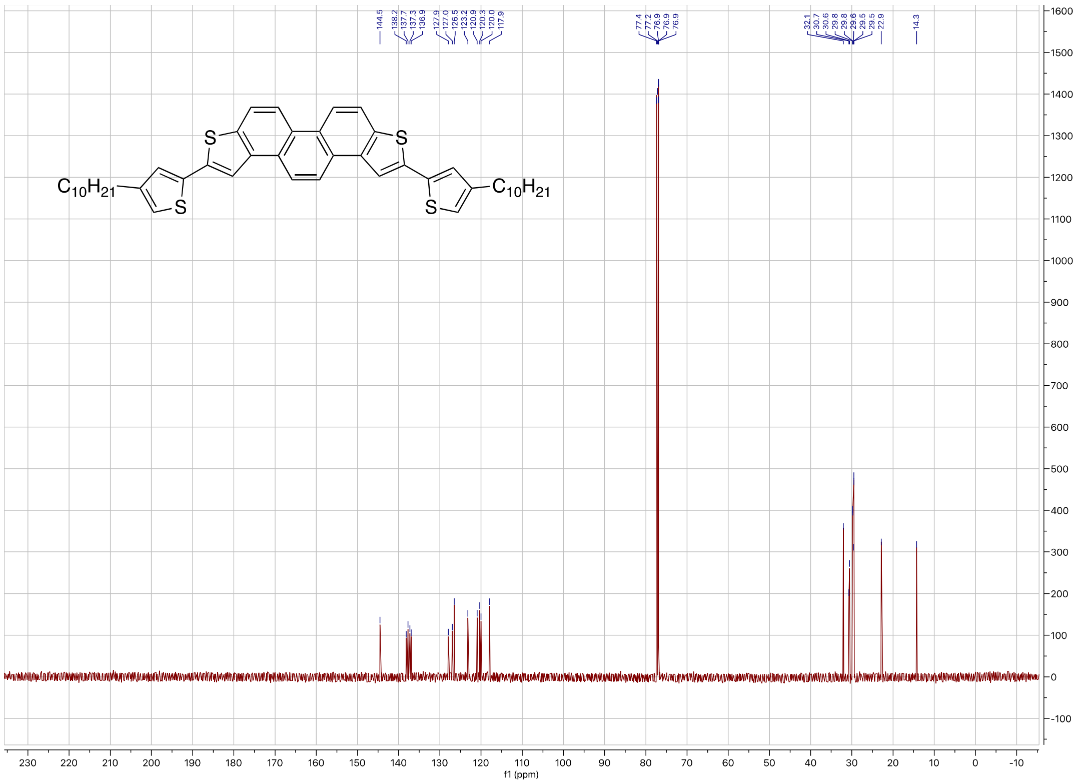

^{13}C{^{1}H} NMR (150 MHz) spectrum of Th2-PDT-2 ($CDCl_3$, rt).

Table S6. Calculated energies and correction values to thermal energies of stationary points (a.u.).

Structure	SCF done: *E* (RB3LYP)	Zero-point correction	Thermal correction to energy	Thermal correction to enthalpy	Thermal correction to Gibbs free energy
C_{10}-PDT-2	-2274.60126704	0.792480	0.834975	0.835920	0.706593
Th1-PDT-2	-3378.21679876	0.886742	0.938639	0.939583	0.785153
Th2-PDT-2	-3378.21589138	0.886589	0.938358	0.939302	0.785886

Table S7. Cartesian coordinates for optimized structures of C_{10}-PDT-2

C	-6.205991491	-5.070988813	0.77201408
C	-4.902392822	-5.413485687	1.166014919
C	-4.027291687	-4.39435249	1.660116423
C	-4.498106907	-3.050547118	1.74249587
C	-5.831607637	-2.769053613	1.326951889
C	-6.67647844	-3.748146594	0.850830005
C	-2.693774623	-4.694451888	2.072231815
C	-3.608604426	-2.013725176	2.244596403
C	-2.284530093	-2.363039708	2.643342274
C	-1.859583829	-3.722232027	2.542373072
C	-1.409685871	-1.34276958	3.135524456
C	-1.871529404	-0.019124299	3.216838618
C	-3.176025822	0.331856621	2.826197952
C	-4.016000052	-0.652636823	2.352535727
C	-2.33892341	-5.718348465	2.008585696
C	-6.210366285	-1.755425289	1.380277951
C	-7.687517047	-3.498814543	0.542317782
C	-0.851235668	-3.984498077	2.846973522
C	-3.518922043	1.360055089	2.895297297
C	-5.01749638	-0.364469108	2.056373893
C	-4.650536443	-6.820126262	0.993758276
C	-0.048875714	-1.456724512	3.590377868
C	0.50513932	-0.280025237	3.999613956
C	-0.637324843	1.054270179	3.844423056
C	-7.093213681	-6.46999222	0.202045323
C	0.50717094	-2.38766776	3.618012094
C	-3.708404787	-7.302730964	1.230587567
C	-5.707367433	-7.521030167	0.492893148
C	-5.788594064	-8.993858429	0.206879087
C	-6.093166933	-9.156955584	-0.836887675
C	-4.776993888	-9.409622359	0.300783586
C	1.897800997	-0.041756694	4.510872862

C	1.858294712	0.4627768	5.486877069
C	2.361414952	-1.020714952	4.688947724
C	-6.746810701	-9.768359704	1.135513673
C	-7.75907048	-9.351823564	1.042382286
C	-6.442754662	-9.603952928	2.177993993
C	-6.780684748	-11.27163707	0.832538028
C	-7.074268362	-11.42486333	-0.216702023
C	-5.765513417	-11.68526846	0.927222528
C	-7.734200683	-12.05426202	1.745338983
C	-8.748672657	-11.63960439	1.650489373
H	-7.440546048	-11.90022084	2.794193956
C	-7.771313873	-13.55861976	1.444622087
H	-8.063696902	-13.71180542	0.395071233
H	-6.756605533	-13.97313122	1.540039702
C	-8.726037058	-14.34184712	2.355576287
H	-9.740452752	-13.92670212	2.260373208
H	-8.433537401	-14.188775	3.405088948
C	-8.764363225	-15.84610908	2.054756435
H	-9.056337474	-15.99887166	1.004968153
H	-7.749897671	-16.26131497	2.150315579
C	-9.719545753	-16.62967445	2.964644309
H	-10.73441739	-16.21514421	2.869531262
H	-9.428033756	-16.47792984	4.014809465
C	-9.758673176	-18.13391869	2.664070986
H	-10.05055194	-18.28572474	1.615031223
H	-8.744912662	-18.54847003	2.759852203
C	-10.71514504	-18.90843564	3.577130638
H	-11.7437086	-18.53991643	3.477431311
H	-10.72015992	-19.97807108	3.337621877
H	-10.42838563	-18.80436337	4.630996935
C	2.79052642	0.779360447	3.557218413
H	2.326848763	1.759107122	3.378568753
H	2.827084635	0.275485321	2.582013916
C	4.212954406	0.975215751	4.096798105
H	4.165210022	1.471503101	5.0776044
H	4.673017024	-0.008051467	4.276144147
C	5.111569004	1.791621073	3.158239314
H	4.651002913	2.774447657	2.979326018
H	5.157517816	1.295320207	2.177603111
C	6.536027714	1.989159254	3.693784861
H	6.489561839	2.485684033	4.674503103
H	6.995934334	1.005919234	3.873462025
C	7.435254247	2.804851131	2.755262635
H	6.975048933	3.787870151	2.575468959

H	7.48132583	2.308225405	1.774592132
C	8.859934714	3.002960439	3.289878616
H	8.813730226	3.499808455	4.2705142
H	9.319945911	2.019795881	3.470000086
C	9.759498405	3.818163993	2.351543836
H	9.30032858	4.801743311	2.171062409
H	9.806565982	3.321759075	1.370612615
C	11.18432296	4.016762581	2.88546833
H	11.13755366	4.513447207	3.865182787
H	11.64352982	3.034131782	3.065200486
C	12.07554426	4.831756077	1.942121826
H	11.66026972	5.832687156	1.770739959
H	13.08485141	4.955753579	2.351306788
H	12.17010973	4.342313825	0.964777992

Table S8. Cartesian coordinates for optimized structures of Th1-PDT-2

C	-5.396122093	-2.86593606	0.707318642
C	-4.00976765	-2.799690824	0.484751827
C	-3.397839223	-1.527761114	0.239172288
C	-4.209832291	-0.355430415	0.225497957
C	-5.608674663	-0.49182535	0.45818542
C	-6.200690412	-1.714251013	0.695522327
C	-1.994890222	-1.409988062	0.008056903
C	-3.589287087	0.937123726	-0.023801182
C	-2.182646043	1.003424722	-0.249302543
C	-1.412840169	-0.197582771	-0.226038043
C	-1.572702271	2.276469026	-0.494044372
C	-2.372219569	3.432514607	-0.507210303
C	-3.758406634	3.372952552	-0.286497366
C	-4.342155724	2.146246451	-0.050687491
H	-1.379634343	-2.304105386	0.018453184
H	-6.24456513	0.385198335	0.452112903
H	-7.270504329	-1.781643558	0.869620463
H	-0.342442551	-0.143839917	-0.397659577
H	-4.362836088	4.275075815	-0.300276275
H	-5.411908707	2.119493037	0.118073666
C	-0.195709389	2.580929131	-0.748232243
C	0.056861606	3.915093122	-0.94617892
C	-4.277806919	-5.116347269	0.810009051
C	-3.399435861	-4.094552606	0.550643702
S	-5.930371198	-4.510780383	0.980327702
S	-1.424927942	4.871643613	-0.818688141
H	-2.337289828	-4.27020035	0.423734053
H	0.589935723	1.835535481	-0.795896825
C	1.558730848	5.875778878	-1.578887654
C	2.937352299	6.171529132	-1.779790805
C	1.318826501	4.567241306	-1.224498088
C	3.769130813	5.099282277	-1.583956049
C	-4.233042857	-8.823708809	1.39037178
C	-4.841045011	-7.535775562	1.38389284
C	-2.931409577	-8.825056044	0.959424214
C	-4.007291431	-6.532015912	0.944971336
S	-2.437250123	-7.2021466	0.524494963
S	2.837704597	3.686477668	-1.132709249
H	-4.750685466	-9.720771708	1.710570269
H	-5.861692218	-7.356072439	1.70561011
H	0.767386719	6.607895601	-1.702618439
H	3.296347379	7.153943396	-2.064755824
C	5.268214445	5.007667997	-1.703636046

C	5.952013631	6.335313919	-2.061133525
H	5.528803252	4.254623244	-2.462040967
H	5.687689841	4.631667306	-0.758924553
H	5.550982687	6.707451478	-3.013740034
H	5.702711896	7.089243523	-1.30208973
C	7.476992799	6.203080115	-2.167708865
C	8.177143043	7.521935374	-2.521291445
H	7.724774108	5.445383884	-2.925706535
H	7.876756854	5.823508934	-1.215778464
H	7.776579034	7.901930307	-3.47282077
H	7.928368722	8.279233151	-1.763107003
C	9.702594536	7.394326509	-2.628479327
C	10.40425645	8.713288226	-2.97878091
H	9.951083577	6.637915147	-3.387770553
H	10.1023771	7.01189089	-1.677502834
H	10.00411708	9.095989447	-3.92953204
H	10.15547725	9.469386693	-2.219233345
C	11.92975862	8.586313435	-3.086017498
C	12.6322057	9.905181844	-3.434350491
H	12.17845736	7.830882117	-3.846332298
H	12.32954031	8.202329377	-2.135571531
H	12.23314126	10.28997203	-4.384904795
H	12.38437754	10.66111643	-2.67408617
C	14.15770416	9.778838407	-3.541793141
C	14.85097158	11.10053686	-3.88943328
H	14.40553552	9.024330087	-4.302059776
H	14.55664362	9.394821042	-2.592010524
H	15.93789371	10.97704932	-3.958873456
H	14.49804654	11.49210278	-4.851624414
H	14.65051801	11.86530323	-3.128750932
C	-1.960812203	-9.969952938	0.828276257
C	-2.545600861	-11.33620239	1.214864859
H	-1.074782909	-9.770689982	1.449021884
H	-1.590741175	-10.01843194	-0.206411736
H	-2.900780734	-11.30016378	2.253766027
H	-3.425659542	-11.54524186	0.591554877
C	-1.530387613	-12.47666475	1.062968894
C	-2.099499933	-13.84985057	1.443943302
H	-0.647827571	-12.26389649	1.68415715
H	-1.170946435	-12.50798367	0.023801284
H	-2.459070432	-13.8180984	2.483149847
H	-2.982922297	-14.0611498	0.823333692
C	-1.088390496	-14.99414028	1.292268948
C	-1.657479395	-16.36792715	1.671225557

H	-0.205213716	-14.78294774	1.913416026
H	-0.72796327	-15.02452604	0.253231765
H	-2.017834205	-16.33740869	2.710315315
H	-2.541032614	-16.57835084	1.050300437
C	-0.647473807	-17.51312435	1.519038634
C	-1.21661018	-18.88707248	1.896655834
H	0.235866763	-17.30298869	2.140454734
H	-0.286631343	-17.54297456	0.480043744
H	-1.577354815	-18.85829509	2.935837317
H	-2.100068869	-19.09797099	1.275461442
C	-0.207455837	-20.03295459	1.744363942
C	-0.784424021	-21.4011554	2.123195548
H	0.674910524	-19.82289671	2.365663983
H	0.15268102	-20.06219074	0.70602275
H	-0.040323355	-22.19710415	2.003815672
H	-1.121301966	-21.41393202	3.167245399
H	-1.647447626	-21.65501138	1.495158023

Table S9. Cartesian coordinates for optimized structures of Th2-PDT-2.

C	-4.488306893	-5.05527199	-0.224323781
C	-3.093012343	-5.03514578	-0.054521071
C	-2.388803321	-3.792849935	-0.170209014
C	-3.120096252	-2.602036834	-0.45433953
C	-4.533100289	-2.69100405	-0.614151034
C	-5.213701847	-3.884911987	-0.504844189
C	-0.973280545	-3.722747456	-0.006560487
C	-2.40590262	-1.339696856	-0.572664165
C	-0.990225928	-1.320979362	-0.402177783
C	-0.303282733	-2.538594827	-0.117745137
C	-0.288112924	-0.077666121	-0.519641903
C	-1.007675372	1.096747382	-0.800770277
C	-2.402594592	1.083870949	-0.969997037
C	-3.075204755	-0.114072625	-0.855336927
H	-0.419775431	-4.630467906	0.211893171
H	-5.10866559	-1.79893062	-0.829798037
H	-6.291548394	-3.916328468	-0.633594785
H	0.773936556	-2.520681428	0.013869723
H	-2.944766613	1.999432691	-1.187053809
H	-4.150325476	-0.104854344	-0.988027012
S	0.043651015	2.493326954	-0.894965563
C	1.116895835	0.177402028	-0.398296614
C	1.464604258	1.492619237	-0.574083629
C	-3.52615335	-7.328267231	0.25289427
C	-2.572403308	-6.343200016	0.21363041
S	-5.140181536	-6.669052197	-0.036429975
H	-1.519682996	-6.559374972	0.355638768
H	1.857741223	-0.589899821	-0.204584818
C	2.781161587	2.096029978	-0.523162482
C	-3.353780961	-8.747045247	0.492713976
C	3.178743725	3.339808051	-0.95852989
C	4.568576536	3.620630012	-0.762107374
C	5.212142828	2.564715121	-0.170027664
C	-4.237587682	-9.776030015	0.259070079
C	-3.741993843	-11.07356607	0.605307348
C	-2.467210514	-11.00379722	1.105063483
S	-1.863235549	-9.377729373	1.171350443
S	4.147653845	1.234643287	0.163067854
H	-5.222113643	-9.616291847	-0.17046625
H	-1.838653827	-11.81485206	1.445532806
H	2.496264634	4.041646731	-1.428595383
H	6.25655564	2.484887946	0.097590968
C	5.205015978	4.928361961	-1.176910685

C	6.706637317	5.051602116	-0.891764873
H	4.675111602	5.752308056	-0.676107035
H	5.029378333	5.08061581	-2.252301192
H	6.886417893	4.914183882	0.18386159
H	7.243138688	4.239173312	-1.401968916
C	7.289963776	6.399944792	-1.334257774
C	8.792073388	6.534993801	-1.051468862
H	6.750270967	7.213508302	-0.827254116
H	7.108721997	6.539232603	-2.410335886
H	8.972520089	6.394024058	0.024615386
H	9.331397828	5.721166722	-1.558630783
C	9.3796932	7.882697841	-1.490892869
C	10.88150333	8.01751406	-1.205960519
H	8.839537939	8.696489434	-0.984491315
H	9.200097336	8.023527914	-2.567165981
H	11.06057245	7.875631957	-0.129704308
H	11.42146336	7.203699022	-1.712595322
C	11.47070261	9.365121728	-1.643610426
C	12.97208282	9.49991223	-1.357306982
H	10.93027241	10.17895313	-1.137417502
H	11.29236682	9.506862444	-2.720045239
H	13.15115924	9.358217493	-0.280868501
H	13.51343465	8.686619551	-1.863638028
C	13.56243015	10.84730682	-1.793908471
C	15.06186988	10.97306804	-1.503610841
H	13.02204319	11.66002734	-1.287775475
H	13.38461696	10.98865905	-2.869596548
H	15.26780796	10.87075738	-0.430846876
H	15.63311804	10.19468837	-2.02467388
C	-4.554736753	-12.33526595	0.41892474
C	-3.859869443	-13.63687457	0.83644696
H	-4.85250287	-12.40962903	-0.637630119
H	-5.495819801	-12.23416194	0.979856963
H	-2.924552883	-13.74949342	0.270152279
H	-3.575078517	-13.57526007	1.896302336
C	-4.736328352	-14.87719767	0.61846273
C	-4.052848784	-16.1867526	1.033698502
H	-5.023260054	-14.93758502	-0.441840788
H	-5.674671191	-14.76290351	1.181188571
H	-3.113167329	-16.29937681	0.472698942
H	-3.767514277	-16.12663847	2.094525226
C	-4.925912718	-17.42933912	0.813593604
C	-4.243267948	-18.73919627	1.229551234
H	-5.210616348	-17.48934982	-0.247451397

H	-5.866142089	-17.31615527	1.373621835
H	-3.302282642	-18.85140465	0.670539171
H	-3.959653203	-18.67943083	2.290923847
C	-5.115053455	-19.98239086	1.007710819
C	-4.433017145	-21.29229194	1.423904796
H	-5.398289723	-20.04216064	-0.053810049
H	-6.056299714	-19.86996952	1.566317441
H	-3.491414433	-21.40543097	0.865812488
H	-4.150145488	-21.23372825	2.485707014
C	-5.303885617	-22.53598849	1.201564189
C	-4.614900648	-23.83937468	1.619740631
H	-5.586033453	-22.59504691	0.140613852
H	-6.244384674	-22.42353615	1.759713239
H	-5.261737045	-24.70763071	1.44878748
H	-3.688614078	-23.99739673	1.053419603
H	-4.351732289	-23.82457679	2.684738895
H	15.45266911	11.94498625	-1.826322174
